# Hypertension in Young Adults: Treat It Before It’s Too Late

**DOI:** 10.7759/cureus.72758

**Published:** 2024-10-31

**Authors:** Manish Gaba, Naveen Kumar, Ankita Pandey, Arun Dewan

**Affiliations:** 1 Internal Medicine, Max Smart Super Speciality Hospital, New Delhi, IND

**Keywords:** hypertensive emergency, iga nephropathty, secondary hypertension, severe hypertension, young people

## Abstract

Hypertension affects a major part of the world population and is one of the leading modifiable risk factors for cardiovascular disease. Its prevalence is increasing in all age groups due to various factors, which include poor lifestyle. Among young people, it poses a special challenge and needs critical reappraisal. In this group, there is a lack of awareness of the disease, poor compliance with treatment, and underdiagnosed secondary causes. An interesting case, where a young man with a diagnosis of hypertension for three years, who was not complying with the physician’s treatment advice, came to us with a hypertensive emergency with end-organ damage. We try to touch on all these important issues with this report.

## Introduction

Hypertension is one of the most common ailments that we see in our daily clinical practice. Patients presenting in hypertensive emergencies are very frequent in the emergency department. If this presentation is seen in a young individual in the age group of 20-40 years, it requires a detailed workup for secondary causes. A detailed counseling of the patient is necessary to ensure compliance with treatment. We attempt to impress the distinctness of hypertension in young patients and discuss the associated aspects with an interesting clinical case.

## Case presentation

A 27-year-old male patient came with complaints of blurry vision and floaters in the eye for three days. The patient had no history of diplopia, neck pain, weakness in any limb, slurring of speech, altered sensorium, seizure, head trauma, fever, hematuria, or drug abuse. He gave no history of cardiovascular disease or connective tissue disorder in the past. He was a known smoker (four pack-years) and was diagnosed with essential hypertension during a medical checkup at his office three years back. He was advised to undergo a complete workup at that time. However, he never started treatment and did not undergo any investigations. The patient had initially visited the emergency department of another hospital with these symptoms and was found to have elevated blood pressure of 260/155 mmHg. His blood investigation done at the other center revealed elevated serum creatinine of 2 mg/dl and the ultrasound abdomen was suggestive of medical renal disease. When we examined the patient, his blood pressure was 200/140 mmHg and 196/140 mmHg in the left and right arms, respectively, in the supine position. In the standing position, it was 180/130 mmHg and 176/130 mmHg in the left and right arms, respectively. The patient’s heart rate was 90/minute, all peripheral pulses were palpable, no carotid or renal bruit was heard, and there was no radio-radial or radio-femoral delay. The patient’s fundoscopy was done, which revealed grade IV hypertensive changes in both eyes with papilledema and macular exudates in the right eye. The patient’s general physical examination and systemic examination were within normal limits.

Investigation

The patient's initial blood work (Table 1) revealed deranged renal functions with a serum creatinine of 2.4 mg/dl (creatinine clearance = 54) with normal serum electrolytes. His liver function tests, lipid profile, and complete blood count were normal. Serum albumin was 3.35 gm/dl, suggestive of mild hypoalbuminemia. The patient’s urine routine revealed blood 1+, protein 3+, 7-10 red blood cells/high power field (RBC/hpf), 1-2 leucocytes/hpf, and no casts. The twenty-four-hour urine protein was in the nephrotic range with 6 gm of protein. His autoimmune profile and anti-streptolysin O (ASO) titer were negative. The C3 and C4 complement levels were within normal limits. The patient’s glycated hemoglobin and serum thyroid stimulating hormone were normal. He tested negative for hepatitis C, hepatitis B, and human immunodeficiency virus I and II.

**Table 1:** Laboratory investigations SGOT: Serum glutamic oxaloacetic transaminase; SGPT: Serum glutamate pyruvate transaminase; ALP: alkaline phosphatase; GGT: gamma-glutamyl transferase; TSH: thyroid stimulating hormone; iPTH: intact parathyroid hormone; ESR: erythrocyte sedimentation rate; HBsAg: hepatitis B surface antigen, HCV: hepatitis C virus; HIV: human immunodeficiency virus; RBC: red blood cell; ANA: Antinuclear antibody; IF: Immunofluorescence; ASO: Antistreptolysin O; Anti-DS DNA: Anti-double stranded DNA; ANCA: Antineutrophil cytoplasmic antibodies; GBM: glomerular basement membrane

Investigation	Value	Reference range
Hemoglobin (g/dl)	14	13-17
Total leucocyte count (cell/cum)	9400	4-10
Platelet (cell/cumm)	225	150-410
Creatinine (mg/dl)	2.4	0.9-1.3
Sodium (mmol/L)	135	136-146
Potassium (mmol/L)	4.6	3.5-5.1
Calcium (mg/dl)	8.8	8.8-10.2
SGOT (IU/L)	26	15-41
SGPT (IU/L)	43	17-63
ALP (IU/L)	78	32-91
GGT (IU/L)	30	7-50
Albumin (g/dl)	3.35	3.5-5
HbA1c (glycated hemoglobin)	4.8	<6.5
TSH (uIL/ml)	1.03	0.3-5.6
iPTH (pg/ml)	56	12-88
ESR (mm/hr)	6	0-10
HBsAg	Negative	
HCV IgG	Negative	
HIV-I&II	Negative	
	Urine analysis	
Blood	2+	Nil
Protein	2+	Nil
RBC (cell/cumm)	7-10	0-1
Ketone	Nil	Nil
Glucose	Nil	Nil
Leucocyte (cell/cumm)	1-2	0-5
Cast	Nil	Nil
Epithelial cell	0-1	0-1
pH	6.5	5-6
Specific gravity	1.012	
24-hour urine protein (mg/dl)	5992.19	40-150
	Autoimmune profile	
ANA-IF	Negative	
ASO (IU/ml)	<100	0-200
Anti-DS-DNA- IF	Negative	
C3 (mg/dl)	150	79-152
C4 (mg/dl)	35	16-38
c-ANCA	Negative	
p-ANCA	Negative	
Anti-GBM antibody	Negative	

**Figure 1:**
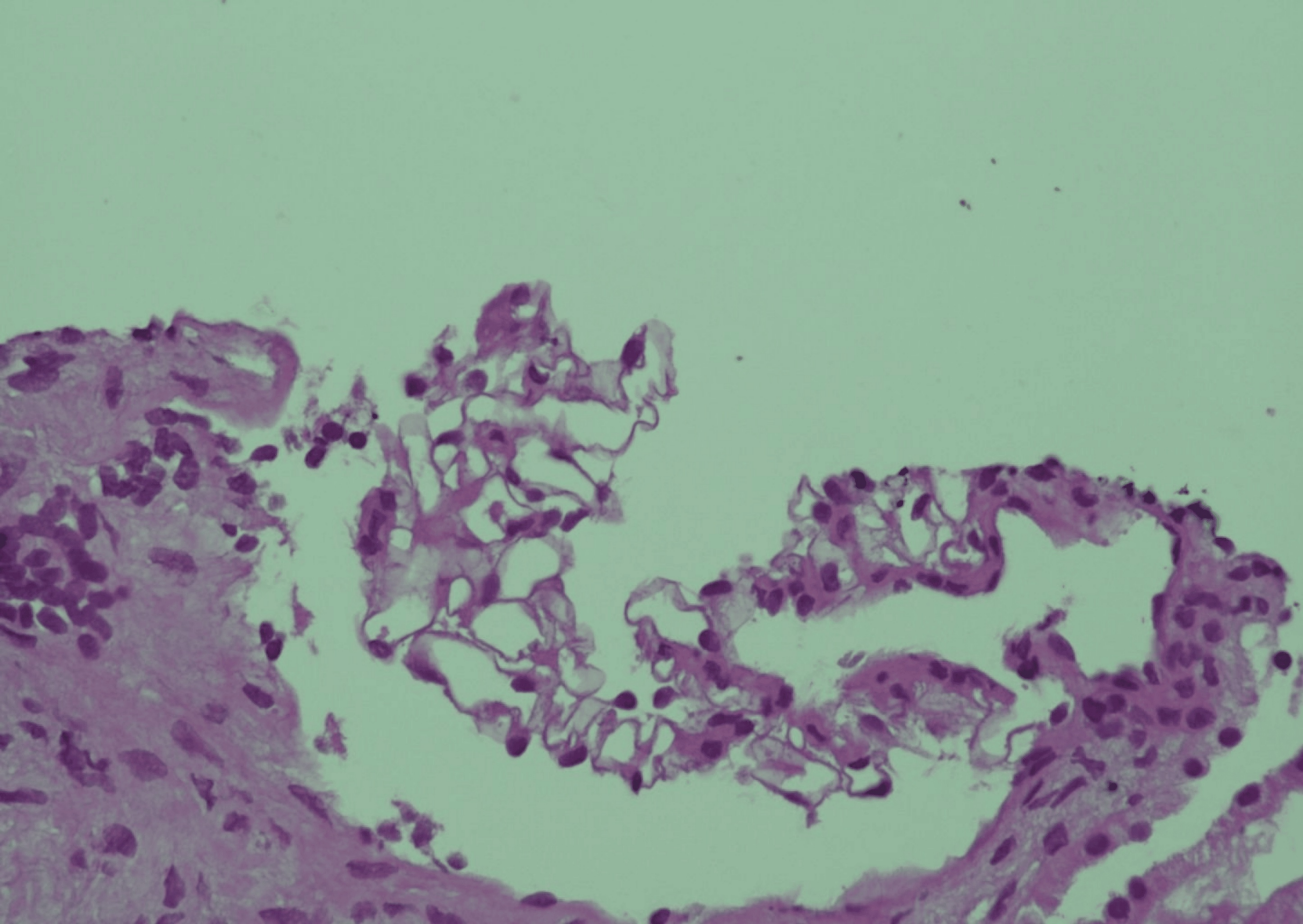
Histopathology of renal biopsy suggestive of IgA nephropathy (H&E, 400x) Glomerular capillary walls of normal thickness with patent capillary lumens, which are not bloodless or sclerosed, are present, the mesangium shows segmental increased cellularity, and endocapillary proliferation is not seen. ATI: Acute tubular injury; IFTA: Interstitial fibrosis and tubular atrophy; IgA: Immunoglobulin A

**Figure 2:**
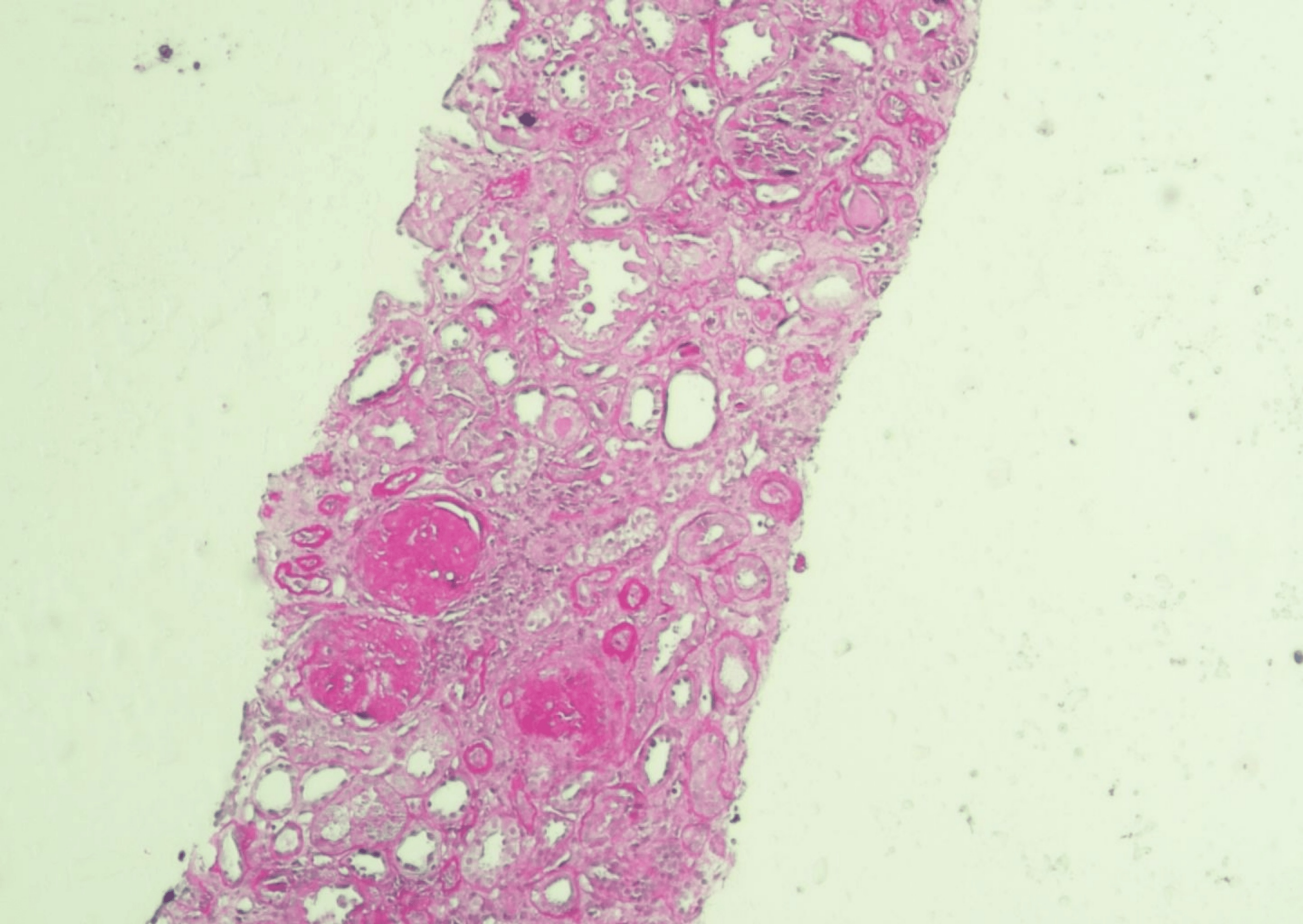
Histopathology of renal biopsy suggestive of IgA nephropathy (H&E, magnification 100x) In total, 66% of glomeruli are globally sclerosed, no fibrinoid necrosis is seen, and one glomerulus shows fibrocellular crescent. Tubulo-interstitium shows mild ATI, interstitium shows mild lymphoplasmacytic infiltrate in areas of IFTA, IFTA is 15%, and no vasculitis is present. IgA: Immunoglobulin A; ATI: Acute tubular injury; IFTA: Interstitial fibrosis and tubular atrophy

The patient’s renal artery Doppler revealed no renal artery stenosis and ultrasonography of the abdomen revealed normal renal size with increased cortical echogenicity. The investigation done to this point did not reveal a diagnosis. The only positive findings were deranged renal function, proteinuria, and increased cortical echogenicity on the ultrasound abdomen. The case was reviewed with our nephrology team and it was decided to perform a renal biopsy. The patient's renal histo biopsy revealed Immunoglobulin A (IgA) nephropathy (Figures 1, 2). The Oxford classification was M1 E0 S0 C1 T0 with 66% global glomerulosclerosis and 16.6 % fibrocellular crescent. There was no fibrinoid necrosis or evidence of vasculitis.

**Figure 3:**
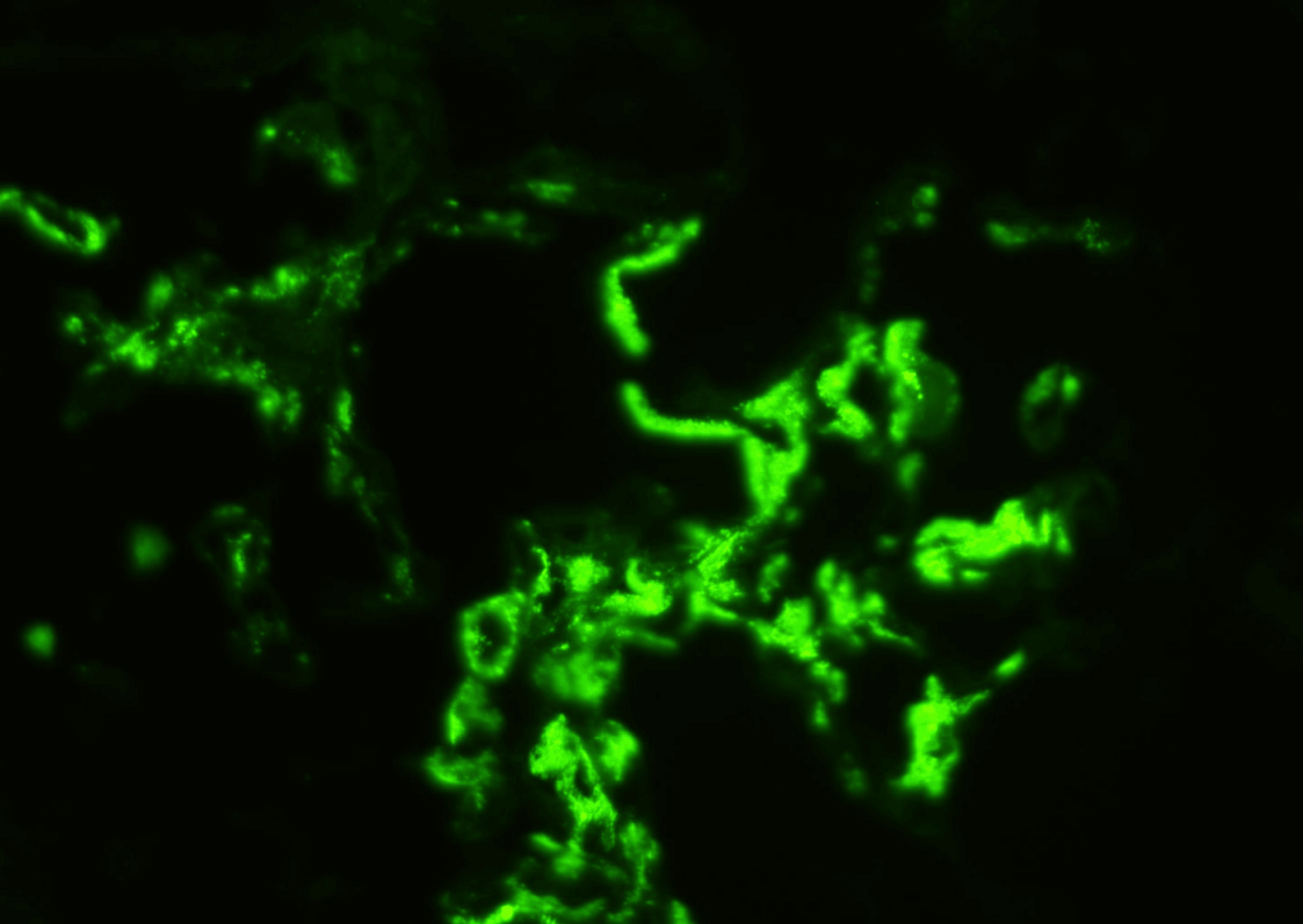
Immunofluorescence microscopy demonstrating mesangial immunoglobulin A (IgA) deposits diagnostic of IgA nephropathy

The sections were 3+ for mesangial positivity for IgA and 1+ for mesangial positivity for C3 (Figure 3). Stainings for IgG, IgM, and C1q were negative. The patient's PLA2 receptor antibody was negative. 

Differential diagnosis

The patient is a young individual who presented with a hypertensive emergency. His workup was done for secondary causes of hypertension. His lab work revealed elevated creatinine levels and nephrotic range proteinuria. There was no evidence of renal artery stenosis with a negative immunology workup. The patient was suspected to have nephrotic syndrome. In consultation with the nephrology team, the patient's renal biopsy was done, which revealed IgA nephropathy.

Treatment

The patient was started on labetalol infusion for high blood pressure, and as it gradually reduced, he was shifted to oral anti-hypertensives (amlodipine, clonidine, and metoprolol). The patient was started on IV steroids. Our patient was initially managed as a hypertensive emergency and gradually shifted to angiotensin-converting enzyme (ACE) inhibitors and other oral antihypertensives. He was started empirically on IV steroids in consultation with the nephrologist in view of the acute glomerular disease. The patient was discharged on oral steroids.

Follow-up

On follow-up, his renal function was improved, visual symptoms were settled, and blood pressure was fairly controlled. Steroids were stopped and antihypertensive medicines were continued.

## Discussion

One in eight individuals in the age group of 20-40 years all over the world has hypertension, which is expected to increase due to poor lifestyle behavior and the hypertension diagnosis threshold going down [1]. In a study from South Africa, Jones et al. found the prevalence of hypertension in the age group of 25 to 34 years increasing from 15% to 33% and 10.6% to 27% in males and females, respectively, from the year 1998 to 2016 [2]. A study conducted in Kerala (India) showed that among the young adults, 40.8% were obese, 58.5% had abdominal obesity, 44.9% had hypercholesterolemia, 6.9% had diabetes, 17.7% had impaired fasting glucose, 96.7% had low intakes of fruits and vegetables, 23.7% of the men were smokers, and 19.4% of the men had consumed alcohol during the previous year. These are important social risk factors that are in play in young individuals in India [3].

In spite of the fact that timely diagnosis and treatment of hypertension in young individuals are even more important due to the cumulative effect of sub-clinical organ damage over time, they are diagnosed and treated much later for various reasons: lower awareness about hypertension, young individuals make infrequent visits to primary care clinic, and they are often negligent about health due to pre-occupation with work [1,3,4]. In a systemic review conducted by Devi et al. in India, they concluded that the control of hypertension is suboptimal, and awareness about it is low [5]. Studies have found underutilization of and poor compliance with preventive medicines in young patients. According to a study by Mehta et al., compared to people of age >65 years, those of 35-44 years of age are 30-40% less likely to be on recommended cardio-vascular medicines dispensed to them [6]. In another study, Hashmi et al. concluded that young age and poor disease awareness are associated with poor adherence to anti-hypertensive treatment [7]. Our patient clearly supports these conclusions. He is a well-educated man of middle socio-economic status and despite being diagnosed with hypertension for three years and recommended by doctors for follow-up medical checks, he never followed the advice and came into hypertensive crisis with end-organ damage.

Although essential hypertension is the primary cause of hypertension in young patients, secondary hypertension is more common in this group. In a study by Camelli et al., the prevalence of secondary hypertension in young hypertensive patients less than 40 years old was about 30% [8]. The chance of a secondary cause is higher if blood pressure remains uncontrolled on multiple medications, accelerated hypertension is episodic, and the patient presents in a hypertensive crisis. Among secondary causes, renal diseases are the most common in young individuals. In our patient, the significant proteinuria and microscopic hematuria with very high blood pressure were indicative of renal involvement, and a clinical diagnosis of nephritic syndrome was made. The diagnosis was however only confirmed by renal biopsy as IgA nephropathy. 

IgA nephropathy is described as one of the most common forms of glomerulonephritis in the world. There is a male predominance in prevalence and a peak incidence is observed in the second and third decades of life. It is an immune complex-mediated glomerulonephritis, defined by the presence of diffuse mesangial IgA deposits and associated with mesangial hyper-cellularity. The diagnosis can only be confirmed by a renal biopsy. The recently described Oxford classification has helped standardize the pathologic characterization of IgA nephropathy using a scoring system that is readily reproducible and associated with increased risk of glomerular filtration rate (GFR) loss independent of clinical variables. 

The most common clinical presentation of IgA nephropathy, reported in 40-50% of patients, is macroscopic hematuria, usually following an upper respiratory tract infection, accompanied by proteinuria. In other 30-40% of patients, it presents with asymptomatic microscopy hematuria or proteinuria during routine evaluation. In less than 10% of patients, it has nephrotic syndrome, acute rapidly progressive glomerulonephritis, and malignant hypertension. A hypertensive emergency is an uncommon presentation of IgA nephropathy, which carries a worse prognosis for renal outcomes [9]. In a retrospective study by Jaryal et al., 93.4% of patients had blood pressures of more than 140/90 mmHg, but malignant hypertension was reported in only 3.3% of them [10]. In another series by Sevillano et al. studying malignant hypertension in IgA nephropathy patients, its prevalence was 7% (13/186), with remarkably worse renal outcomes [11]. The renal survival in cases of IgA nephropathy with and without malignant hypertension has been summarised in Table 2.

**Table 2:** Renal survival in patients of IgA nephropathy compared with that of patients of IgA Nephropathy with malignant hypertension Adapted from [11-12] IgA: Immunoglobulin A

Disease	Five-year renal survival	Ten-year renal survival
IgA nephropathy	84%	72%
	Three-year renal survival	Six-year renal survival
IgA nephropathy with malignant hypertension	69%	35%

All patients in the series by Sevillano et al. had renal failure at admission [11]. Interestingly, like in our patient, none of the patients had been previously diagnosed with IgA nephropathy, nor had been studied for suspected kidney disease. Moreover, despite good blood pressure control, six months from the time it was achieved, all showed progressive deterioration of renal function, which continued till the rest of the follow-up. They concluded that in patients with malignant hypertension with hematuria significant proteinuria and high serum IgA levels, one must suspect and rule out IgA nephropathy. In conditions with nephrotic range proteinuria, serum albumin of >3.5 gm/dl can predict IgA nephropathy with a specificity of 95.8% [13]. Although nephrotic-range proteinuria is not uncommon in IgA nephropathy, the coexistence of nephrotic syndrome is rare.

IgA nephropathy constitutes 7% to 16% of most biopsy samples in India [9]. Studies show that IgA nephropathy has a more severe presentation and rapid progression in people from South or East Asian regions compared to any other origin [9]. In a study from India of 478 patients, Chacko et al. reported renal survival of 84%, 55%, and 33% at one, five, and 10 years, respectively. [14] In a study from Canada, Barbour et al. found that the risk of developing end-stage renal disease (ESRD) was significantly higher in people from the Pacific Asia region compared to North America (hazard ratio: 1.56, 95% confidence interval: 1.10-2.22) [15]. Risk factors for rapidly developing ESRD were hypertension, male sex, older age of onset, nephrotic proteinuria, persistent hematuria, interstitial fibrosis, and sclerosed glomeruli.

A unique feature of IgA nephropathy compared to other glomerular diseases is that the GFR loss starts at a much lower level of proteinuria (protein excretion: 1 g/day) [16]. So, treatment targets are to reduce proteinuria < 1 g/day and the resolution of microscopic hematuria. The use of ACE inhibitors (ACEI) and angiotensin receptor blockers (ARB), at the maximum recommended doses, has an important role as it controls blood pressure as well as reduces proteinuria. However, there are certain limitations. The use of ACEI is associated with dry, nonproductive paroxysmal cough, and there is no treatment for the cough. This significantly limits their use. Angioedema is an important side effect of ACEI, which can affect any part of the body. This can cause airway obstruction, which can be life-threatening [17]. Angioedema has a higher rate of incidence in the African-American population. ACEI can cause hyperkalemia [17]. The risk for this side effect is more common in individuals with a history of renal impairment, diabetes, and simultaneous use of potassium-sparing diuretics. The incidence of angioedema and cough with ARBs is less than with ACEI and they are better tolerated [18]. These drugs are contraindicated in patients with bilateral renal artery stenosis or patients with heart failure who have hypotension.

Additional supportive care includes other antiproteinuric or renal protective therapies (mineralocorticoid receptor antagonist, sodium-glucose co-transporter 2 inhibitors), and lifestyle modification (smoking cessation, weight control, exercise, and dietary sodium and protein restrictions). After three months of supportive care, if proteinuria of >1 g/day persists, patients are categorized as high-risk and are offered immunosuppressive therapy. In individuals who present with ESRD, there is no role of steroids or immunosuppressive therapy.

Our case highlights important issues related to young hypertensive patients. Despite being hypertensive for three years, the patient was not convinced enough that the hypertension should be treated and had poor compliance with treatment and follow-up. It can be counted as a fault on the part of the treating physicians also that even after knowing that he was young and a secondary cause was a possibility, he was not counseled enough to ensure adherence to detailed evaluation and treatment. Moreover, as it turned out to be a secondary treatable cause, all end-organ damages were preventable. Hence, it is important to do thorough workups of hypertensive emergencies in young patients.

## Conclusions

Hypertension in young patients is a special situation. Patients in this group are mostly not convinced by the diagnosis and don't feel the need for treatment. Secondary hypertension is common in this group, which can be difficult to treat. Treatment of hypertension in young individuals is even more important due to the cumulative effect of sub-clinical organ damage over time. An early diagnosis and treatment can have a marked effect on the prognosis of a patient with IgA nephropathy and can prevent ESRD with the early institution of the appropriate therapy.
